# From Botox Party to Botulism: A Case Report and Public Health Warning on Adulterated Botulinum Toxin Injection

**DOI:** 10.7759/cureus.68016

**Published:** 2024-08-28

**Authors:** Emily F Dawson, Soniya Pateriya, Zachary A Blashinsky, Amalia Landa-Galindez

**Affiliations:** 1 Internal Medicine, Herbert Wertheim College of Medicine, Florida International University, Miami, USA

**Keywords:** botulism antitoxin, iatrogenic botulism, botox parties, botulism, counterfeit botox

## Abstract

Botulinum toxin (BoNT) is a potent neurotoxin with broad applications in medicine and cosmetics. Cases of botulism toxicity from aesthetic injections have been spiking in recent months across the United States. Here, we present a patient who presented with bulbar paralysis and increasing respiratory distress following BoNT injections performed at her home by an unlicensed aesthetician. The patient presented with ptosis, dysphagia, dysarthria, shortness of breath, and proximal muscle weakness. The patient was given botulism antitoxin and showed marked improvement in the following days. The pathophysiology of iatrogenic botulism is unknown but may be due to improper injections with large doses or direct injection into the bloodstream. Counterfeit BoNT injections have been reported and may include unsafe components or dangerous levels of BoNT that surpass the therapeutic dose.

## Introduction

Botulinum toxin (BoNT), a neurotoxin produced by the bacterium *Clostridium botulinum*, paralyzes skeletal muscle by inhibiting the release of acetylcholine [1]. There are seven distinct BoNT types (A-G), of which types A, B, E, and, rarely, F cause human botulism, a potentially life-threatening condition causing neuroparalysis with multisystem involvement [1,2]. Botulism can take six clinical forms: foodborne botulism, wound-associated botulism, infant botulism, adult intestinal toxemia, inhalation botulism, and iatrogenic botulism [3].

Iatrogenic botulism is a rare and severe complication of administering BoNT for medical or non-medical purposes. Symptoms of botulism vary but can include dysphagia, ptosis, and diplopia, as well as more severe presentations that may include systemic weakness, orthostatic hypotension, gastrointestinal delayed emptying or paralysis, or muscle paralysis [1]. Diagnosis is usually based on the exclusion of other neurologic disorders, history of botulism exposure, clinical presentation, and neurologic findings on examination. Rapid diagnosis is essential, as delayed administration of botulinum antitoxin can worsen the outcome. Cerebral spinal fluid analysis and brain imaging are usually ordered to exclude other causes. Electromyography (EMG) is nonspecific but may show an increment in compound motor nerve action potential amplitude, decreased recruitment of muscle units, and decreased duration of muscle unit potentials. EMG may not be readily available at all hospitals and requires expert interpretation [1].

According to a report of Centers for Disease Control and Prevention (CDC) data, prior to 2024, the last reported case of iatrogenic botulism in the United States was in 2017, following intravenous drug use [4]. Other cases have developed after using OnabotulinumtoxinA (Botox®; Allergan Inc., Irvine, CA, USA) for medical treatment, such as hyperhidrosis [5,6]. Botox was first approved by the Food and Drug Administration (FDA) in 1989 for treating strabismus, blepharospasm, and hemifacial spasm. The cosmetic use of BoNT for facial rhytides became more commonplace, and in 2002, the FDA approved Botox for the treatment of glabellar lines, crow’s feet, and forehead lines [7].

In April 2024, the FDA announced multiple reports of individuals contracting botulism after receiving BoNT. Individuals from Colorado, Florida, Illinois, Kentucky, Nebraska, New Jersey, New York, Tennessee, and Washington reported botulism-like symptoms after receiving what was likely adulterated BoNT products [8].

Medical practices buy BoNT products, like Botox, which licensed practitioners safely administer to patients. However, unintended consequences may arise when BoNT products are administered without careful patient selection, dosing calculations based on specific muscle groups, and precise injection techniques to only the intended areas. There is an increased risk of adverse outcomes when BoNT products are obtained from questionable sources or administered by unlicensed practitioners.

Herein, we present a case of botulism contracted from the improper use of BoNT, involving a likely counterfeit product.

## Case presentation

A 59-year-old female with a past medical history of hypertension and hypercholesterolemia presented to the Emergency Department with shortness of breath, trouble speaking, blurry vision, and numbness of the throat for one week. The patient stated that her symptoms began after receiving “Botox” for cosmetic purposes to her forehead, bilateral temples, glabella, nose, mouth, and chin. The injection was administered at her house during a “Botox party” by an unlicensed individual. She had previously received Botox yearly without adverse side effects. The patient denied any immediate post-injection complications, and no other participants reported adverse effects.

However, four days after the injection, she developed blurry vision in both eyes, accompanied by drooping eyelids, which were more severe on the left than on the right eyelid. Seven days after the injection, speaking and swallowing became more difficult. In addition to her initial symptoms, she began to experience shortness of breath and generalized weakness, particularly in the proximal upper extremities. No underlying neuromuscular disorders had previously been diagnosed in the patient or her family. Further questioning revealed that the patient had not been exposed to potential neurotoxins, including marine mammal meat, salmon eggs, and self-canned goods. She denied a recent or remote history of smoking or intravenous drug use. A review of systems was negative for chest pain, fever, recent infection, skin lesions, nausea, or vomiting.

On physical exam, she was in no acute distress and oriented to person, location, time, and situation. Evaluation of cranial nerves was significant for bilateral ptosis (CN III) and bilateral facial paralysis with garbled speech. Her facial sensation was intact. Muscle strength was diminished bilaterally in the upper and lower extremities (3/5), and she had reduced neck flexion (4/5). She had 2+ reflexes throughout symmetrically; the Babinski reflex was negative bilaterally. Muscle tone and deep tendon reflexes were normal.

Further workup included an electrocardiogram, brain computed tomography, chest X-ray, and urinalysis, all of which were within normal limits. The patient’s vital capacity was continuously monitored to assess for respiratory distress. Various botulism-specific panels and myasthenia gravis labs were collected, as summarized in Table 1. Repetitive stimulation of the left abductor brevis using surface electrodes did not show abnormalities.

**Table 1:** Test results summary

Test	Value	Normal range	Results
Acetylcholine receptor binding antibodies	0.33	Negative: ≤0.30 nmol/L; Equivocal: 0.31-0.49 nmol/L; Positive: ≥0.50 nmol/L	Equivocal
Acetylcholine receptor block antibody	<15%	Negative: 0-26% blocking; Equivocal: 27-41% blocking; Positive: 42% or greater blocking	Negative
Acetylcholine receptor module antibody	<1	Negative: <1	Negative
Striated muscle antibody	<1:40	Negative: <1:40	Negative

Comprehensive diagnostic testing for myasthenia gravis, including assays for acetylcholine receptor (AChR) antibodies and striated muscle antibodies, yielded negative results. Additionally, repetitive nerve stimulation revealed no abnormalities consistent with myasthenia gravis. These findings suggest that this patient's case was not likely complicated by an underlying neuromuscular disorder, such as myasthenia gravis.

A diagnosis of iatrogenic botulism was made due to the evidence of flaccid paralysis, cranial nerve palsies, and lack of sensory deficits in the setting of the recent botulism injection. With a high degree of clinical suspicion of botulism, the medical team administered botulism antitoxin per the CDC recommendations. The patient showed noticeable improvement in her weakness within 24 hours of administering the antitoxin, with almost total resolution by 72-96 hours. As the patient continued to improve and with no concerns for decompensation, she was discharged with scheduled outpatient follow-up with neurology.

## Discussion

Since its approval in 1989, BoNT has proved to be a vital neuromuscular treatment for medical and cosmetic conditions. A myriad of medical BoNT therapies have been approved for cosmetic and non-cosmetic purposes, including chronic migraine, spastic disorders, cervical dystonia, and detrusor hyperactivity [9]. BoNT tends to stay localized after injection but can affect non-targeted muscles or glandular tissue. Complications of locally injected BoNT include ptosis, bruising, ectropion, diplopia, asymmetric appearance, and epiphora [10]. More serious side effects of BoNT, such as neutralizing antibodies, allergic reactions, and botulism, can develop with lower concentrations, thereby increasing diffusion [10]. Lower concentrations, in combination with non-BoNT substances, allow the BoNT to spread to undesired areas. The recent spike in botulism-like cases from adulterated BoNT products provides a cautionary tale regarding the safety of Botox and other BoNT treatments.

When botulism develops, affected individuals may begin to experience nonspecific symptoms, such as nausea, sore throat, abdominal pain, dizziness, and dry mouth [3]. As botulism progresses, cranial nerve paralysis with diplopia, blurred vision, ptosis, dysphasia, dysarthria, and a suppressed gag reflex can develop concurrently with symmetrical descending paralysis or weakness [3]. If botulism affects respiratory muscles, respiratory failure may ensue [3]. Our patient exhibited cranial nerve palsies (ptosis, blurred vision) as well as descending paralysis in the setting of a recent BoNT injection.

The signs and symptoms can be nonspecific at the beginning of the disease course. Clinicians must exclude other causes of neuromuscular paralysis, such as Guillain-Barré syndrome, Miller Fisher variant, myasthenia gravis, Lambert-Eaton syndrome, organophosphate and carbamate poisoning, tick paralysis, snake venom, drugs, hypocalcemia and hypermagnesemia, and shellfish and puffer fish intoxication [11]. Our patient denied a history of neuromuscular disease and the abovementioned exposures. Her recent injection with BoNT, with the subsequent development of blurred vision, ptosis, flaccid paralysis, and respiratory complaints, was highly suggestive of botulism.

Iatrogenic botulism remains a rare cause of botulism, with infant botulism accounting for 60-70% of the over 200 cases reported annually to the CDC [4]. However, several BoNT injection-related cases have been reported over the past two decades. In 2006, Chertow et al. reported four instances of laboratory-confirmed botulism [12]. All four patients had received BoNT type A from an unlicensed preparation; three of the four patients were found to have serum toxin levels 21 to 43 times the estimated human lethal dose. A Chinese case series analyzed 86 patients from 2009 to 2013 who developed botulism after being treated with BoNT for cosmetic purposes [13]. A nursing student who purchased and injected a bottle labeled "Thigh Slimming Injection" developed botulism after unknowingly injecting her thighs multiple times with BoNT type A [14].

Non-cosmetic use of BoNT has also led to iatrogenic botulism. Ghasemi et al. reported two cases of botulism after hyperhidrosis treatment in 2012 [5]. Rouientan et al. reported a 22-year-old male undergoing Botox treatment for axillary hyperhidrosis who developed botulism in 2019 [6]. As BoNT continues to be the mainstay for multiple cosmetic and medical conditions, we may encounter more unfortunate cases of botulism. Reports of iatrogenic botulism outbreaks outside the United States have been reported, including a series of isolated cases in Europe in 2023. In 2017, a serious outbreak of iatrogenic botulism occurred in Egypt related to an unlicensed preparation sold as Neuroxin, which affected nine patients receiving intramuscular injections for the treatment of cerebral palsy, spastic dystonia, and hyperhidrosis. All patients required hospitalization but fortunately fully recovered within 6-12 weeks [15].

Treatment for botulism is centered around supportive care, respiratory support to prevent respiratory failure, and administration of antitoxin. In 2013, the FDA approved the first botulism antitoxin that can neutralize all seven known botulinum neurotoxin serotypes [16]. The antitoxin is best delivered within 24 hours of symptom onset, as it can neutralize circulating toxins, but it cannot reverse paralysis that has already developed [17].

Shortly after the admission of our patient, the CDC released a statement regarding adverse reactions linked to counterfeit "Botox" across the United States. As of April 18, 2024, a total of 22 individuals reported symptoms similar to those seen in botulism toxicity [8]. Further information was provided on 20 individuals: 55% were hospitalized, and 27% received the botulism antitoxin due to concern for systemic spread. Of the seven people tested for botulism, six received negative results, and the final individual is still pending results. All individuals reporting symptoms are female, with a median age of 41. In recent observations, 91% of people reported receiving BoNT injections for cosmetic purposes, and notably, all reported receiving the injection in a non-healthcare setting by unlicensed or untrained individuals. This underscores the importance of utilizing products from authorized sources and administration by licensed and thoroughly trained medical professionals.

**Figure 1:**
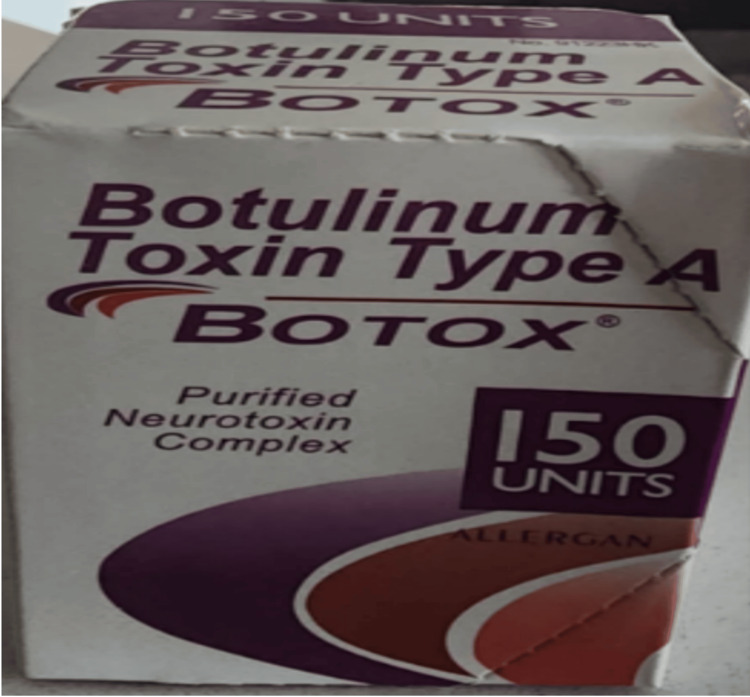
Counterfeit Botox outer packaging Image credit: [18]

A commonality among previous reports [13,14] of botulism toxicity following BoNT administration includes the lack of standards involving medical training, certification, volume, and dosage. We urge policymakers and the healthcare industry to implement greater regulation surrounding this ubiquitous practice for patient safety. We recommend reviewing products utilized and heightened awareness of counterfeit products for consumers. The CDC recommends the potential identification of counterfeit products through the following labels on the outer carton and vial, as illustrated in Figure 1 (Appendices): “The outer carton and vial contain lot number C3709C3; the outer carton displays the active ingredient as ‘Botulinum Toxin Type A’ instead of ‘OnabotulinumtoxinA’; the outer carton and vial indicate 150-unit doses, which is not a unit made by AbbVie or Allergan; and the outer carton contains language that is not English” [18]. 

Our case stresses the importance of diagnosing botulism early in patients presenting with cranial nerve palsies, paralysis, and respiratory complaints, particularly after having received BoNT in the recent past. Respiratory failure is life-threatening, and immediate recognition of botulism can allow for critical supportive care as well as the delivery of antitoxin. Our patient recovered well with treatment and was advised to receive future BoNT from a licensed medical professional. With its widespread use in today’s society, BoNT has extended beyond the walls of a medical professional’s office to personal residences (“Botox Parties”). When BoNT is obtained through the proper channels and injected under appropriate medical supervision, undesired side effects can be mitigated. However, unsupervised use and administration can result in serious consequences. We encourage all providers to counsel patients regarding the potential risks associated with the use of BoNT outside the care of a licensed healthcare provider. Healthcare providers should also be aware of guidelines for reporting suspected botulism cases, and suspected cases should be reported to your local health department. The CDC also offers 24-hour consultation services for botulism cases and any travel-related emergencies at 770-488-7100 [19].

Due to the rise of BoNT injections, particularly from unauthorized sources, there is a greater need for more extensive research into the safety protocols, dosage regulations, and long-term outcomes of BoNT therapy. This case highlights several areas where further studies could be valuable. For instance, investigations into the efficacy of current safety guidelines and training programs for BoNT administration could help refine best practices. Further exploring the pharmacokinetics of BoNT and how it diffuses into non-target tissues at varying concentrations could lead to improved formulations and a reduction of unintended effects. Additionally, conducting regulatory and policy research across countries on preventing the sale and use of counterfeit BoNT products could inform the development of more effective global standards. By expanding the understanding of both the benefits and risks of BoNT, particularly in non-medical settings, future research can contribute to improved patient outcomes and public health.

## Conclusions

Iatrogenic botulism secondary to BoNT injection is a rare complication, with an increasing reported incidence in the United States. Here, we report a patient admitted with bulbar paralysis secondary to botulism toxicity following “Botox” injections by an unlicensed professional. While the pathophysiology of this presentation is not specific, it is likely due to either provider error during the injection process (incorrect technique or excessive non-approved dosing) or a recent rise in counterfeit products that fail FDA-approved standards for cosmetic application. This patient had multiple sites injected on the same day, which increased the risk of complications and potentially serious side effects. Further investigations are necessary to understand the recent spike in such cases, and proper regulations by policymakers to deter unlicensed use of BoNT injections for cosmetic purposes need to be encouraged.
